# GeneXpert for TB diagnosis: planned and purposeful implementation

**DOI:** 10.9745/GHSP-D-12-00004

**Published:** 2013-03-21

**Authors:** Amy S Piatek, Maarten Van Cleeff, Heather Alexander, William L Coggin, Manuela Rehr, Sanne Van Kampen, Thomas M Shinnick, YaDiul Mukadi

**Affiliations:** a United States Agency for International Development, Washington, DC, USA; b KNCV Tuberculosis Foundation, The Hague, Netherlands; c Centers for Disease Control and Prevention, Division of Global HIV/AIDS, Atlanta, GA, USA; d U.S. Department of State, Washington, DC, USA; e Centers for Disease Control and Prevention, Division of Tuberculosis Elimination, Atlanta, GA, USA

## Abstract

*Xpert MTB/RIF* is a major advance for TB diagnostics, especially for multidrug-resistant (MDR) TB and HIV-associated TB. But implementation concerns including cost, technical support requirements, and challenging demands of providing second-line TB drugs for diagnosed MDR-TB cases call for gradual, careful introduction based on country circumstances.

## INTRODUCTION

Tuberculosis (TB) continues to be one of the greatest killers in the world due to infectious disease, claiming over 1.4 million deaths in 2011.^1^ In recent years, the prevention, diagnosis, and treatment of TB has become more complicated because of 2 factors changing the epidemic: HIV-associated TB and multidrug-resistant (MDR) TB. Many people die from TB because their diagnosis is delayed, and the epidemic continues to endure because we are unable to significantly reduce transmission with current diagnostics. *Xpert MTB/RIF®* (or *Xpert*), based on the GeneXpert platform, offers a major breakthrough against these limitations—but only if it is implemented within a context of strong national program and laboratory strategic plans and according to a comprehensive technical approach that includes everything from planning to evaluation.

## BACKGROUND

Sputum smear microscopy remains the most common way to diagnose pulmonary TB. Depending on the report and method used, smear microscopy can accurately detect TB in 20% to 80% (using fluorescence microscopy methods) of TB cases.^2^ Sputum smear microscopy has significant limitations because it can only be used to diagnose TB when sputum has sufficient bacillary load, and it cannot detect drug resistance. Thus, HIV-associated TB often goes undetected because people living with HIV (PLHIV), especially those with severe immunosuppression, generally have very low numbers of bacilli.^3^

A more sensitive approach to diagnosis is to culture sputum samples, which can include testing for drug resistance. However, such techniques require expensive and sophisticated laboratory infrastructure and staff, and it can take weeks or months to obtain results. Realistically, most people who need culture tests to diagnose their TB will not have access to the test results in time to save their lives or to prevent transmission to others.

With the advent of new molecular diagnostics, a rapid and sensitive test to diagnose TB, including HIV-associated TB and MDR-TB, is within reach. The *Xpert MTB/RIF* assay from Cepheid, Inc., is a molecular-based rapid test with potential to revolutionize TB diagnosis. However, a key question looms large: Do resource-constrained countries have the technical and financial resources to appropriately and adequately implement this new test? If so, how should they proceed?

## WHAT IS XPERT MTB/RIF?

The *Xpert MTB/RIF* assay is a fully automated molecular diagnostic test for TB disease developed in partnership among Cepheid, Inc., the Foundation for Innovative New Diagnostics (FIND), the University of Medicine and Dentistry of New Jersey (UMDNJ), and the National Institutes of Health (NIH). It can simultaneously detect *Mycobacterium tuberculosis* (MTB) complex DNA and mutations associated with rifampicin (RIF) resistance (a reliable proxy for MDR-TB) directly from sputum specimens in less than 2 hours, and it minimizes staff manipulation and biosafety risk.^4^

*Xpert* can detect TB, including MDR-TB, in less than 2 hours, potentially reducing the time to diagnose and treat TB.

*Xpert* is more sensitive than sputum smear microscopy in detecting TB, and it has similar accuracy as culture.^5-6^ Moreover, its ability to detect smear-negative TB provides a significant advantage, especially for PLHIV. Importantly, its ability to detect RIF-resistant TB in less than 2 hours significantly improves the likelihood of timely treatment initiation. (Conventional culture and drug-susceptibility testing [DST] are still required to complete the drug-resistance profile and to monitor treatment.)

*Xpert* does cost more than smear microscopy; it requires a machine that currently costs US$17,000 and cartridges that cost US$9.98 for each test, in addition to human resource and other running costs. It also has operational limitations, such as the need for a sustained power supply. However, *Xpert* is intended to be used at facilities close to the patient to reduce the time to diagnosis and TB treatment initiation.

## XPERT AS GLOBAL POLICY

In December 2010, the World Health Organization (WHO) endorsed *Xpert* for the rapid and accurate detection of TB, particularly among PLHIV and people suspected of having MDR-TB.^7^ The global TB community responded to quickly roll out and scale up *Xpert* in high TB-burden countries by developing policies, guidelines, and monitoring frameworks to support Ministries of Health (MOHs) in their implementation.

In December 2010, WHO endorsed *Xpert* for detecting TB.

## IMPLEMENTATION REQUIREMENTS

Performing the *Xpert* assay is relatively simple and involves minimal specimen manipulation. However, the numerous operational and programmatic requirements associated with the assay and its results cause implementation to be less easy than expected. Priority requirements include both operational and programmatic considerations.

**Operational requirements:**

- Uninterrupted power supply
- Ambient temperature no higher than 30°C
- Biosafety equivalent to smear microscopy
- Adequate storage for test kits (or cartridges) at temperatures no higher than 28°C
- Waste disposal system for cartridges
- Secure location to protect machine and computer from theft
- Trained laboratory and clinical staff
- Annual calibration of the *Xpert* modules

**Programmatic requirements:**

- Review (and revision) of diagnostic algorithms, policies, forms, and guidance
- Capacity for conventional culture and drug-resistance testing through diagnostic referral networks
- Quality-assured microscopy network to monitor drug-sensitive TB treatment
- Capacity for MDR-TB treatment, including facilities, staff, and drugs
- Computer and software technical support
- Inventory and supply chain management for commodities
- Routine monitoring, evaluation, and supervision of implementation
- Budget to support initial investment of machines and infrastructure and to support running costs for cartridges and calibration

**Additional technical requirements:**

- Coordination mechanisms in country, and epidemiological and SWOT (Strengths, Weaknesses, Opportunities, and Threats) analysis of diagnostic and treatment situation to guide implementation
- Integrating *Xpert* into national laboratory strategies for both the public and private sectors and country plans for initial implementation, including identifying target groups, defining diagnostic algorithms, selecting appropriate sites, forecasting commodities, and developing an annual activity plan and budget
- Ensuring infrastructure and operational needs are met to begin *Xpert* testing at designated sites
- Building capacity for *Xpert* implementation, including training of site staff and clinicians
- Monitoring routine *Xpert* implementation and evaluating the impact of roll out

## COORDINATED COUNTRY SUPPORT

In response to WHO's endorsement and technical assistance needs, the United States Government (USG) (including the Centers for Disease Control and Prevention [CDC], the Office of the Global AIDS Coordinator [OGAC], and the United States Agency for International Development [USAID]) is supporting implementation and impact-assessment projects to facilitate in-country *Xpert* introduction and scale up in a systematic, phased, and coordinated manner. To maximize the impact, these projects support not only machine and cartridge procurement but also the MOHs, by providing comprehensive technical assistance toward operational and programmatic requirements. Additionally, the USG supports research studying different implementation models and their potential impact on TB care and management programs, including transmission and mortality.

Planning and carrying out activities according to the operational and technical requirements mentioned above is necessary but can be challenging and potentially demanding on countries with limited resources.

## KEY CHALLENGES AND LESSONS LEARNED

Cost and infrastructure requirements are key challenges to *Xpert* implementation. Efforts to date have identified many other challenges and lessons learned.

### Costs and Sustainability

The 4-module *Xpert* machine currently costs approximately US$17,000. The cost of one test cartridge is US$9.98, which was recently reduced by 40% (from US$16.87) through a financial agreement with the manufacturer and the Bill & Melinda Gates Foundation, PEPFAR, UNITAID, and USAID. The actual cost per test will vary by country because of differences in shipping fees, procurement agent and other clearance fees, and the use of required distributers. A reduced pricing scheme for the machine and cartridges for the public sector was negotiated with Cepheid in 145 high TB-burden countries.^8^ As seen with other sophisticated test systems (for example, CD4 count tests for HIV), additional cost cuts for automated nucleic acid amplification tests such as *Xpert* may occur as competing technologies enter the market.

In total, first-year initial investment costs, including associated commodities (such as the machine, cartridges [3,000/machine/year at full capacity], uninterrupted power supply, and printer), calibration, and other human resource needs are estimated at US$61,000. Annual running costs for cartridges and calibration are estimated at about US$32,000 per machine.

The price of *Xpert* equipment and cartridges is a barrier for scaling up *Xpert* in many countries. In countries that already have *Xpert* machines, we fear that the machines will sit unused after the initial investment unless due attention is given to identifying sustained resources for commodities and recurrent costs.

Equipment and supply costs are a barrier to scaling up *Xpert* in many countries.

Countries also need to factor in the cost of treatment for each MDR-TB case detected by *Xpert*. The cost of drugs for treating an MDR-TB case is 50 to 200 times greater than treating a drug-sensitive TB case, and the overall costs to care for each MDR-TB case are 10 times higher.^9^ Many countries do not currently have the financial resources to treat their existing MDR-TB patients, and the detection of additional cases by *Xpert* is likely to further strain such health systems.

### Prioritizing According to Country Circumstances

When, where, and how to use *Xpert* depends on the national commitment to draft policies and implementation strategies; available funds; accessibility, availability, and geographic distribution of adequate diagnostic services; and the epidemiology of TB in the country (especially HIV-associated TB and MDR-TB). Positioning of *Xpert* machines in the country needs to balance available resources, national capacity building, and accessibility to persons suspected of having TB that would most benefit from the diagnostic test.

Because it is a new and expensive technology, many countries are placing their first machines in central- and regional-level labs to gain knowledge, build a cadre of staff who can provide technical assistance on the assay, and most importantly, test as many people suspected of having TB as possible. Given limited resources, *Xpert* should be targeted to at-risk populations, particularly those with suspected HIV-associated TB and/or MDR-TB, to produce a high yield and high impact of early diagnosis. In addition, many countries continue to do parallel diagnostic smear microscopy to preselect persons suspected of having TB and build the local evidence base, but also because their national policies to treat and monitor TB patients rely on smear microscopy status.

### Testing and Treatment Algorithms

*Xpert* should be incorporated into a diagnostic and treatment algorithm that includes all diagnostic tests needed to place a patient on an adequate drug regimen. In some settings, such as among populations with a low frequency of MDR-TB or high frequency of RIF-resistant TB, this may include confirmation of RIF resistance or MDR-TB by conventional culture and DST.

### Diagnosing and Treating MDR-TB

Many countries currently have limited ability to address drug-resistant TB. Lack of TB culture and DST facilities and referral systems continues to delay diagnosis and treatment. Drugs for MDR-TB are available, but they are expensive and often require injections and up to 2 years of treatment. Similarly, weak MDR-TB treatment capacity, including facilities and staff, means that confirmed drug-resistant cases may go untreated until these systems are strengthened. This poses a substantial dilemma for countries who must weigh the benefits of diagnosing MDR-TB against the ethics of not being able to provide sufficient treatment. Increased capacity to detect MDR-TB should dictate that countries and their partners significantly ramp up treatment capacity.

Countries must be prepared to treat the increased MDR-TB cases that *Xpert* detects, with drugs costing 50 to 200 times more per MDR-TB case than for a drug-sensitive TB case.

### Training of Both Laboratory and Clinical Staff

Training has focused on laboratory staff members who operate the machine and perform the assay. However, clinical staff members need to be sensitized to *Xpert*, so that they properly use the results to inform treatment. Often, clinicians continue to want smear, culture, and drug-sensitivity test results, even in the presence of an *Xpert* test result. Clinicians and medical associations need to be included in *Xpert* stakeholder meetings and trainings.

### Delayed Turnaround Time of Test Results

Although *Xpert* test results can be ready in 2 hours, many people receive their results days later, often due to laboratory operations issues, including limited staff, practices of batching specimens, and other logistical barriers such as inefficient specimen referral and transport networks. The promise of detection within 2 hours or on the same day is achievable but may be challenging because of these barriers.

### Technical Support Needs

The *Xpert* assay is a computer-based test. Many facilities have limited technical support to help overcome problems encountered with either the hardware or software. These problems can mean that a simple “glitch” can translate into days or weeks of downtime. Language is also another barrier for many countries because the software is currently only in English. Adequate, readily available technical support is needed, in addition to building capacity within the National TB Reference Laboratory system to address potential bottlenecks and technical issues related to implementation.

### Monitoring and Evaluation

A robust monitoring and evaluation system needs to be put in place, including appropriate indicators and support for data collection, reporting, and analysis. It is especially important to monitor the positive effects that *Xpert* can have on treatment initiation rates and reduced time to treatment. Assessment of these effects requires a system that can link diagnostic and clinical information, which is not yet in place in most high-burden countries.

## OPPORTUNITIES

Despite the challenges to implementing *Xpert*, a number of opportunities for strengthening many aspects of TB prevention, diagnostic, and treatment programs are emerging.

### Public-Private Partnerships

The successful public-private partnership among Cepheid, FIND, NIH, and UMDNJ to develop the *Xpert* assay may serve as a model for other collaborations to develop even better diagnostics and new technologies for TB. Similarly, the donor partnership between the Bill & Melinda Gates Foundation, PEPFAR, UNITAID, and USAID immediately increased affordability of *Xpert* cartridges.

### Synergy With HIV Efforts

Since use of the machine in HIV-treatment settings to diagnose co-infected TB patients is highly recommended, *Xpert* scale up in these settings may strengthen the coordination between TB and HIV programs. Although policies to intensify TB case detection among persons with HIV have long been in place, a significant gap remains in on-the-ground collaborative activities. *Xpert* has the potential to narrow this gap. Additionally, implementation of *Xpert* can be greatly facilitated by leveraging HIV laboratory, care, and treatment infrastructure. TB programs can garner other lessons learned from HIV programs, which have witnessed rapid implementation of new technologies and treatments. In addition, Cepheid and other companies are looking into performing TB testing and HIV viral load testing on a common instrument.

*Xpert* can improve coordination between HIV and TB programs.

### Stimulating Focus on MDR-TB

Since TB programs finally have a tool to quickly diagnose RIF resistance, they need to ensure that the capacity to treat confirmed MDR-TB patients keeps pace with diagnosis. The early detection of MDR-TB cases through *Xpert* must create new treatment sites and strengthen existing policies and guidance to treat MDR-TB patients. Similarly, increased detection of MDR-TB should increase the market for second-line anti-TB drugs and potentially drive the cost of these drugs down.

Increased detection of MDR-TB through *Xpert* could increase the market for second-line anti-TB drugs and drive costs down.

### Strengthening Other Laboratory Diagnostics

There is still a need for high-quality microscopy, culture, and DST to monitor treatment and outcomes and to complete the susceptibility profile. Therefore, *Xpert* roll out should motivate countries to continue strengthening laboratory networks and specimen-referral networks throughout the country to keep up with this demand.

### Creating Strong Platforms for New Innovations

Currently, there are no point-of-care TB diagnostic tools at the stage of evaluation or demonstration that are sufficiently sensitive and specific for TB detection in both populations with and without HIV infection. However, a variety of nucleic acid amplification, alternative antigen, and volatile organic compound detection assays are in the pipeline or are proving to have utility in distinct patient populations.^10^ The introduction of *Xpert* is expected to strengthen health systems and laboratory networks, which will, in turn, help to create platforms that will make it easier to launch these and other future diagnostics and drug therapies. Coordination of *Xpert* roll out by MOHs and partner and stakeholder working groups will build a high level of capacity to prepare for promising upcoming changes in technology and tools.

## CONCLUSION

*Xpert* is the most exciting innovation in TB diagnostics in over a century. It has the potential to significantly increase TB case detection in 2 priority populations in which traditional diagnostics are woefully inadequate—people with suspected HIV-associated TB and MDR-TB. The possibility to diagnose TB in these important groups in 2 hours will lead to fewer deaths and less transmission of disease.

However, *Xpert* is not a panacea. Its implementation presents major challenges, particularly related to cost and infrastructure, which call for a thoughtfully phased and careful introduction. Strong health systems are required in order to realize the full potential of this new technology. Also, *Xpert* is not a point-of-care test, which remains an important need in TB diagnostics. Fast and accurate detection of TB and MDR-TB needs to happen at the community level with a point-of-care test and a strong laboratory network and referral system to ensure that patients have access to all the diagnostic and follow-up testing they need.

Nevertheless, *Xpert* is more than just a “test”—it is transforming the way we think about diagnosing TB. Countries have to make decisions about where to place the test; clinicians have to learn to trust the test results; program managers must embrace the challenges of implementing a new technology; and policy makers must agree to invest with adequate funding for scale up.
